# Preparing for the future of healthcare: Digital health literacy among medical students in Lahore, Pakistan

**DOI:** 10.12669/pjms.40.1.8711

**Published:** 2024

**Authors:** Zobia Farooq, Ahad Imran, Nazish Imran

**Affiliations:** 1 Dr. Zobia Farooq Medical Student, King Edward Medical University/Mayo Hospital, Lahore, Pakistan; 2 Ahad Imran Final Year Computer Sciences Student, Syed Babar Ali School of Science & Engineering (SBASSE) Lahore University of Management Sciences (LUMS), Lahore, Pakistan; 3 Dr. Nazish Imran, MBBS, FRCP, MRCP, MHPE, PhD Professor, Child & Family Psychiatry Department, King Edward Medical University/Mayo Hospital, Lahore, Pakistan

**Keywords:** Digital health literacy, DHL, eHealth literacy, Medical students, Medical education

## Abstract

**Objective:**

Digital Health Literacy (DHL) is becoming a cardinal competence for all healthcare professionals (HCPs) including medical students for meaningful digital transformation of healthcare. As medical students need to navigate an increasingly digitalized healthcare environment, thus the study’s objective was to assess digital health literacy among medical students.

**Methods:**

This cross-sectional analytical study was conducted at King Edward Medical University, Lahore from October 2022 to August 2023. Medical students were asked to complete a pen and paper questionnaire of the Digital Health Literacy Instrument (DHLI) after informed consent. DHLI covers seven categories: operational skills, information searching strategy, analyzing the usefulness of online information, navigational abilities, contributing user-generated material to internet-based platforms, and privacy protection. SPSS was used for data analysis.

**Results:**

Eight hundred ninety one medical students, from first year to final year, participated in the study. The overall mean score for DHL was 63.5 (SD=8.82). Medical students achieved a score of 83.2% of total score in their operational abilities, and 82.3% proficiency in privacy protection, which were deemed highly desirable. Furthermore, they achieved a satisfactory level in navigation skill (76.0%), information searching (73.1%), adding content (71.0%), determining the significance of data (70.1%), and assessing data reliability (68.7%), based on the overall score. A significant relationship was observed between the performance level of DHL domains and gender with higher scores in males in all domains except protecting privacy, which was higher in females and clinical years students (p-value *<* 0.05).

**Conclusion:**

The assessment of the DHL of medical students was deemed desirable. But certain obstacles were encountered in few domains of DHL i.e., data reliability, relevance determination, and content augmentation. It is imperative to elevate the level of DHL of medical students to harness the potential of digital technologies in enhancing healthcare.

## INTRODUCTION

Digitalization in medicine is being considered as a critical answer to the various challenges being faced by healthcare systems all around the globe. These challenges include but are not limited to scarce resources especially in low and middle-income countries (LMICs), shortage of health services workforce, changing demographics of population, rise in non-communicable diseases and comorbidities.^1^

Digital health technologies harbor the potential to transform the health services delivery landscape and enhance outcomes for patients, healthcare workers and health systems. Therefore, the World Health Organization has placed Digital health as a top priority within their digital agenda.^2^ Considering the rapidly evolving state of global health, the widespread accessibility to web-based apps and greater merger between digital technologies and health, it takes a diverse range of skills—referred to as “digital health literacy” (DHL)—to make the most of these opportunities. DHL is described as “the ability to search, find, understand, and evaluate health information from electronic resources and to use the knowledge gained to solve health-related problems”.^3^

DHL is becoming an essential competence for all healthcare professionals (HCPs) for meaningful digital transformation of healthcare. Despite the requirement, existing literature on DHL among healthcare workers is not consistent, with some studies noting that the health care industry is adequately skilled in several DHL areas, including patient privacy protection and operational proficiency,^4^ while other studies found limited DHL of HCPs to act as a barrier to implement digital health services.^5^,^6^ Some challenges to digital health literacy have also been noted, such as users’ lack of the necessary skills in complex online information, online fraud and unverified information, and power outages in low-income nations.^7^-^9^ Various determinants influencing DHL have been identified; Personal determinants (age, gender, ethnicity, level of education), Relational Determinants (Language and cultural barriers), Knowledge determinants (Preexisting knowledge about health issues) and technological determinants like access to devices/ gadgets etc.).^10^-^12^

Medical students, being the future “frontline” workers, need to be prepared with essential skills to navigate an increasingly digitalized healthcare environment.^13^ Studies on DHL of medical students are limited. Studies from Europe noted good DHL in medical students, however difficulties in creating online content and evaluating online information were still faced by many.^14^,^15^ DHL is important not only in service delivery and patient support but also plays a role in medical student’s own health and wellbeing and role as health promoters. Thus, understanding and awareness of level of DHL among medical students can help in guiding curriculum, planning interventions, and addressing it at policy level.^16^

Currently, to our knowledge, there are no studies in Pakistan focusing on DHL among medical students only. Considering the relevance of digital health literacy for medical students joining the health sector, the purpose of this study was to investigate the levels of digital health literacy among undergraduate medical students in Lahore, Pakistan.

## METHODS

In 2023, a cross-sectional analytical study was conducted. Registered undergraduate medical students in all five years at KEMU (a public sector medical school) were eligible to participate. Principal Investigators explained the purpose and significance of the study, its voluntary and confidential nature, and benefits of participation at the beginning of study and participants gave written informed consent. The anonymous pen and paper questionnaire was distributed among all students during lectures. It had two main sections. 1) Personal demographic information. 2) Digital Health Literacy Instrument (DHLI).^17^ The Digital Health Literacy Index (DHLI) was used to assess the digital health literacy of medical students, following communication with the questionnaire authors via email and obtaining their consent. The first five of these categories were graded on a Likert scale of 1–4 (1-very difficult, 2- difficult, 3-easy, and 4-very easy). Navigational skills and protecting privacy were assessed on a four point scale as never-1, 2-sometimes, 3-often, 4-always. The internal consistency of DHLI was measured by Cronbach’s alpha index which was 0.8.

### Ethical Approval

The Institutional Review Board of King Edward Medical University (KEMU) granted ethical approval (112/RC/KEMU).

### Statistical Analysis

After data collection, it was analyzed using SPSS version 26. Descriptive statistics were computed for demographic variables. To compare means between different student groups, analytical statistics (Independent t-test) was used. A p-Value <0.05 was considered statistically significant. Furthermore, the criteria for evaluating each of the seven skills were a percentage of the overall score attained in each skill. Less than 20% score means that skill is ‘very undesirable’, 21-40% means ‘undesirable’, 41-60% means ‘average’, 61-80% means ‘desirable’ and 81-100% means ‘very desirable’.^4^

## RESULTS

Nine hundred fifteen questionnaires were returned out of a total of one thousand distributed to undergraduate medical students (return rate = 91.5%). Because the questionnaires of 24 individuals were incomplete, they were not included in the analysis; hence, the data from N=891 questionnaires was used. Most of them (55% of them) were females. The average age of the students was 21.1 years (standard deviation=1.63). Table-I describes the demographic characteristics of all participants.

**Table-I:** Demographic characteristics of all the participants (N=891).

Category		Number of participants (n)	Percentage (%)
Age in years (mean, SD)	21.1 (1.63)		
Gender	Female	490	55
	Male	392	44
	Prefer not to say.	9	1
Living arrangement	Hostellite	615	69
	Day scholars	271	30.4
Year of Study	First-year	140	15.7
	Second year	312	35
	Third year	112	12.6
	Fourth year	161	18.1
	Fifth year	165	1

The participants’ internet usage trends are shown in Table-II. Of the students, 51.1% reported that they used the internet “almost every day.” Only 101 students claimed to have ‘almost never’ used the internet’s services. The most used device was discovered to be a mobile phone, followed by a laptop, while a public computer was found to be the least liked. The majority of respondents (45.6%) gave themselves “Good” ratings for their internet skills. Numerous internet usage factors that are related to health were also evaluated. The most frequent justification for using the internet was to ‘search information on health and illnesses.

**Table-II:** Pattern of internet usage among the participants. (N=891)

Parameters	Number of participants (n)	Percentage (%)
***Frequency of Internet use***		
(Almost) every day	455	51.1
Several days a week	190	21.3
About once a week	139	15.6
(Almost) never	101	11.3
***Means of Internet access***		
Mobile phone	848	95.2
Laptop	340	38.2
Tablet	89	10
Personal computer at home	57	6.9
Computer at work	26	2.9
Public computer	26	2.9
***Self-rated Internet skills***		
Excellent	173	19.4
Good	406	45.6
Average	241	27
Reasonable	40	4.5
Poor	20	2.2
***Number of respondents who have ever used the Internet to…***		
Search for information on health or illness	809	90.8
Read/post a health relayed review.	625	70.1
Monitor disease symptoms.	605	67.9
Use any health-related app.	531	59.6
Schedule an appointment with their health care provider	283	31.8
Share personal medical information with others.	249	27.9
Ask a question of their health care provider.	240	26.9
Post a message on a peer support forum or social media website.	196	22
Take a web based self-management course.	165	18.5
Log on to their own electronic medical record	153	17.2

The overall mean score for DHL was 63.5 (SD=8.82). It implies that on average, the students manifested a commendable degree of proficiency in digital health literacy. The mean score values of various domains of DHL are shown in Table-III. It shows that the scores of participants in various domains of DHL are ‘desirable’ or ‘very desirable’.

**Table-III:** Digital Literacy Domains and Interpretation.

Domains of Digital literacy	Percentage of Total Score	Mean±SD	Interpretation
Operational skills	83.22	9.98 ± 1.81	Very Desirable
Information searching	73.15	8.77± 1.67	Desirable
Evaluating data reliability	68.78	8.25 ± 1.79	Desirable
Determining data relevance	70.11	8.41 ± 2.10	Desirable
Navigational skills	76.09	9.13 ± 1.72	Desirable
Adding content	71.08	8.53 ± 1.84	Desirable
Protecting privacy	82.37	9.88 ± 1.64	Very Desirable

The independent T-test was utilized to compare the DHLI mean scores and showed significant statistical difference between the two genders. The mean score was higher in males in all DHL domains (p<0.05) other than protecting privacy (p>0.05). There was no statistically significant difference, in mean DHLI scores of students in preclinical years of study than those who study in clinical years in terms of operational and navigational skills, searching information, evaluating data reliability, determining data relevance, and adding content on social media forums (p>0.05), but the mean score for protecting privacy was higher for students studying in clinical years than those studying in preclinical years (p<0.05).

## DISCUSSION

In today’s era of rapidly developing technology and increasing prevalence of digital solutions within the healthcare industry, the value of digital health literacy cannot be overlooked. HCP’s own Digital health literacy is noted to have a significant impact on DHL of patients. Currently, we believe this is the first study in Pakistan to focus on digital health literacy of medical students. The findings indicate that in general, the DHL of medical students is at a desirable level.

Advancements in this field have significant potential that may lead to improved health outcomes and reduce the technology barrier.^18^ Factors such as age, ethnicity, academic background contribute to varying levels of digital literacy across regions of the.^19^ In terms of digital usage patterns among student participants, mobile phones were the most often utilized technical equipment (848, 95%). This discovery is hardly surprising given the swift rise in mobile phone ownership and usage worldwide, including in Pakistan.^20^

The internet is rapidly becoming the primary source of students seeking health information.^21^,^22^ Like previous studies of medical students, study results also indicated searching for information related to health and illness as the most frequent purpose of internet use (91%).^20^ Students medical background can be a contributing factor to the wide gap with only 33%-63% of general internet users using internet for seeking health information.^21^,^22^

Medical students have stronger digital health literacy on average than members of the general public and university students.^23^ DHL demands crucial operational and navigation abilities for searching for and implementing online information in daily life. The current study’s participants received a remarkable 83% of the overall score in the “operational skills” domain; this also being the highest score of all DHL categories and at a highly desired level. Medical students were more likely to navigate the Internet with ease and research on health issues and search with specific keywords (73%). We believe that this could be due to several reasons. A previous study showed that the general public of Pakistan suffers from a lack of access to the latest technologies which contributed to a factor of low digital health literacy.^12^ Medical students, on the other hand, have access to a wide library of academic resources which equip them with up-to-date information on digital health. Digital health topics are also widely integrated into their training which gives them the exposure to be more proficient in using digital health tools. Furthermore, clinical experience helps students to interact with cutting-edge digital technology, increasing their comfort level with these tools. These characteristics offer them far more exposure than the broader population, which might explain these outcomes. It should be noted, however, that while medical majors may have a higher level of digital literacy, this is also reliant on the amount of exposure they receive and their own desire to incorporate it into their everyday practice. A study based in Taiwan also found that Medical Students tend to have greater health concerns which lead to improved eHealth Literacy Development.^24^

Evaluating the quality of health information is an important aspect of health literacy. Unreliable information can have major implications for patient treatment. The greatest obstacles were identified in determining the dependability of data available on the internet and in adding material. We found that while medical students had a desired level of rating the dependability of health information (69%), they had a somewhat wide gap with the desirable level. They were less confident to rely on this information (69%) and use it to take health related decisions for patients. This is in line with other studies where the confidence of reliable information comes into play. A study in Brazil found that students find it difficult to differentiate quality of online resources .^11^ With millions of health-related websites available, it can often become confusing for people to choose a reliable source for their questions.^25^ As the availability of online health information increases, it is imperative to tackle the issue of reliable information and guide people towards approved/validated websites. Promoting awareness and education about reliable health-related sources can assist the public in making informed decisions and avoiding potential hazards connected with misinformation or low-quality resources. This might include identifying reliable sources and fostering critical thinking skills to assess the authenticity of internet health information. Furthermore, students require more supervision and encouragement in terms of adding content as well as their ability to frame health-related queries and communicate their opinions in a comprehensible manner that the recipient of the message may understand.^17^

This study’s findings also revealed that the average DHL of students varied greatly depending on their gender. Male students exhibited higher levels of DHL as compared to females. This aligns with previous studies, which indicate that gender influences one’s ability to assess and evaluate information online.^8^,^26^ As a result, it is critical to devise methods that explicitly target and assist females’ digital health literacy needs to close the gender gap in online health information assessment and evaluation.

### Limitations

Our results are limited by data collection from one Institute, which does not allow generalization of results. Social Desirability Bias is one of the most common sources of research bias. As with most self-reported studies, participants have the tendency to select certain answers which they may believe to be perceived as more desirable. While the study included research design to incorporate self-report measures and maintained anonymity, this could affect the validity of the research. It is recommended that future studies should have both self-report as well as performance-based evaluation methods to reduce bias. Furthermore, given the survey was carried out in the English native language, participants may have misinterpreted certain questions. However, given the fact that medical studies in Pakistan are in the English language, this is not expected to be a significant barrier.

## CONCLUSION

Study contributes to an expanding body of information based on DHL among medical students, who as future healthcare professionals should be able to use technology effectively for better healthcare outcomes. Although overall, DHL of medical students was assessed as desirable, challenges were noted in evaluating reliability, determining relevance, and adding content. It is imperative to increase the DHL levels to a very desirable level in all domains. Study results give medical schools the opportunity for proper policy making and planning for educating and engaging the medical students in improving their DHL skills to an optimal level. Recommendation of incorporating digital health skills explicitly within the medical college curriculum may provide sound basic understanding of digital health and technology assisted care.^27^

### Authors’ contribution:

**ZF** did data collection, statistical analysis and contributed in manuscript writing.

**AI** did literature review and helped with synopsis and writing of manuscript.

**NI** conceptualized the study, supervised the research, did literature review, and critically reviewed the manuscript and is responsible for overall integrity of the work.
